# Exploring strategies to prevent post-lobectomy space: transient diaphragmatic paralysis using Botulinum Toxin Type A (BTX-A)

**DOI:** 10.1186/1477-7800-2-21

**Published:** 2005-10-19

**Authors:** Seyda Ors Kaya, Habip Atalay, Hakan Rıza Erbay, Ali Vefa Özcan, İbrahim Goksin, Burhan Kabay, Koray Tekin

**Affiliations:** 1 Pamukkale University Medical School, Department of Thoracic Surgery, Denizli, Turkey; 2 Pamukkale University Medical School, Department of Anaesthesiology, Denizli, Turkey; 3 Pamukkale University Medical School, Department of Cardiovascular Surgery, Denizli, Turkey; 4 Pamukkale University Medical School, Department of General Surgery, Denizli, Turkey

## Abstract

**Objective:**

Various techniques to reduce air space after pulmonary lobectomy especially for lung cancer have been an important concern in thoracic surgical practice. The aim of this study was to assess the effectiveness of Botulinum toxin A (BTX-A) injection into the diaphragm to reduce air space after right lower pulmonary lobectomy in an animal model.

**Methods:**

Twelve male New Zealand rabbits were randomly allocated into two groups. All animals underwent right lower lobectomy. Then, normal saline of 0,1 ml and 10 units of 0,1 ml Botulinum toxin type A were injected into the muscular part of the right hemidiaphragm in control (n = 6) and BTX-A groups (n = 6) respectively. Residual air space and diaphragmatic elevation were evaluated with chest X-ray pre- and postoperatively. Diaphragmatic elevation was measured as a distance in millimetre from the line connecting the 10th ribs to the midpoint of the right hemidiaphragm.

**Results:**

The mean diaphragmatic elevation in BTX-A and control groups were 7.0 ± 2.5 and 1.3 ± 1.2 millimetres respectively. Diaphragmatic elevations were significantly higher in BTX-A group (p = 0.0035).

**Conclusion:**

Intraoperative Botulinum toxin type A injection may reduce postlobectomy spaces effectively via hemidiaphragmatic paralysis in rabbits. Further studies are needed to validate the safe use of Botulinum toxin type A in human beings.

## Introduction

Lung resection alone is the most effective therapy for the patients with non-small cell lung carcinoma limited to the lung without distant metastasis. Complete resection is the goal of all operations for lung cancer. Every patient with locoregional lung cancer should be approached as a potential candidate for resection. For patients with adequate lung function, the current standard cancer resections include lobectomy, bronchoplastic lobectomy, bilobectomy and pneumonectomy, based on the extent of disease. The appropriate operation depends on the clinical and surgical stage of the tumor. Lobectomy is the ideal operation for resection of a lung cancer confined to the parenchyma of a single lobe[1,2].

After a lobectomy, the pleural space is drained routinely. Approximately 10% of persistent spaces become complicated with empyema or bronchopleural fistula). Lung lobectomies especially in the presence of fibrotic parenchymal disorders often leave a pleural space, and the remaining lobe or lobes may not be sufficient to fill the ipsilateral hemithorax. Residual air space and prolonged air leak after pulmonary lobectomies can potentially cause serious complications such as bronchopleural fistula and empyema, requiring longer hospital stay and increased health costs. Although it is not universally accepted among thoracic surgeons, reducing postlobectomy space could theoretically be useful in preventing the above-mentioned complications. In this respect, several manoeuvres can be used to attempt to decrease the size of the hemithorax including pleural tents, muscle flaps, pneumoperitoneum and phrenic nerve manipulation [1-4].

Botulinum toxin type A (BTX-A) is an extremely potent neurotoxin that interacts selectively with cholinergic neurons to inhibit the presynaptic release of the neurotransmitter acetylcholine [5]. BTX-A is currently used for cosmetic and therapeutic goals for years, but to our knowledge, has not been reported for the treatment of postlobectomy spaces in literature so far. The aim of our present study was to evaluate whether BTX-A can be effective for hemidiaphragmatic paralysis to prevent postlobectomy space in an animal model.

## Materials and methods

The protocol was approved by the Institutional Animal Care and Use Committee, and all animals were housed in the facilities of the Medical Faculty of Pamukkale University.

### Animals

This study was carried out on twelve male New Zealand rabbits weighing between 1.5–2.0 kg. The animals were housed in wire bottom cages at 21–24°C room temperature with 12-hour light dark cycle. All animals were fed on standard laboratory diet and water but received only water for 12 h before surgery.

After an overnight fast, the rabbits were anesthetized by an intramuscular injection of ketamine, 35 mg/kg and xylazine 5 mg/kg. After cardiac monitorization and endotracheal intubation, ventilation was maintained artificially (SAR-830 Rodent ventilator, Geneq Inc., Montreal, Canada).

### Surgical procedure

The right chest wall was shaved, and the animal placed in a left side down position. A skin incision of 3–4 centimetre in length was made on the right anterolateral chest wall under aseptic conditions. The muscles in the 6^th ^intercostal space were bluntly dissected to expose the right thoracic cavity. Right lower lobectomy was performed by ligation of bronchial artery, vein and bronchi with 2/0 silk suture. Then, animals were randomly allocated to two groups. Following right lower lobectomy, the animals in Group 1 (n = 6) served as controls, and 0,1 ml of 0.09% NaCI was injected into the medial and lateral muscular part of the right hemidiaphragm. The animals in Group 2 (n = 6) were injected with 10 U (0, 1 ml) BTX-A (BOTOX^®^, Allergan Pharmaceutical Ltd., Ireland) into the same region as in Group 1. All injections were made using a 26-gauge needle attached to a sterile 1 ml syringe. All animals had a 10F chest tube placed along the diaphragm and for the following two hours aspirated intermittently and chest tube was removed after having negative pressure. Both groups received single dose prophylactic antibiotic, and received diclofenac sodium for postoperative analgesia for 3 days.

### Postoperative period

After the surgery, the rabbits were closely monitored for clinical evidence of pain (vocalisation, tachypnea, and restlessness) for seven days. Chest radiographs were taken for evaluation of diaphragmatic elevation, residual air space and complications. X-rays were taken during in inspiration. Since the onset of paralytic effect of Botulinum toxin type A begins in 2^nd ^day, diaphragmatic elevation was evaluated radiographically in four consecutive antero-posterior-chest radiographs (preoperatively, postoperatively, 3^rd ^and 7^th ^day). All the roentgenograms were taken at a 90 cm distance from the cassette while animals were in erect position. Diaphragmatic elevation was measured as a distance in millimetre from the line connecting the 10^th ^ribs to the midpoint of the right hemidiaphram. Reversal of paralysis was observed by fluoroscopic examination of diaphragmatic movements.

### Statistical analysis

The results are expressed as mean and ± standard deviation (SD). Differences among the groups were evaluated using Mann-Whitney U test. A P value of less than 0.05 was considered significant.

## Results

There were no respiratory distress, prolonged air leak and any other complications requiring treatment in any group. Two animals in BTX-A group had about less than 10% increase in respiratory rate at the 3rd day and normalised at the 4th day (From about 70 to 80 breaths per minutes). There was no changes in dietary patterns, and neither vocalisation nor agitation in any animal.

Immediate postoperative and 3^rd ^day roentgenograms revealed no complications such as hemothorax, mediastinal shift and atelectasis. Minimal residual air space in the base of right hemidiaphragm was detected in 3^rd ^day roentgenograms of two rabbits in BTX-A and three rabbits in control groups. There was no residual air space In BTX-A group but was still remaining in 2 rabbits of control group in 7^th ^day radiographs.

**Figure 1:**
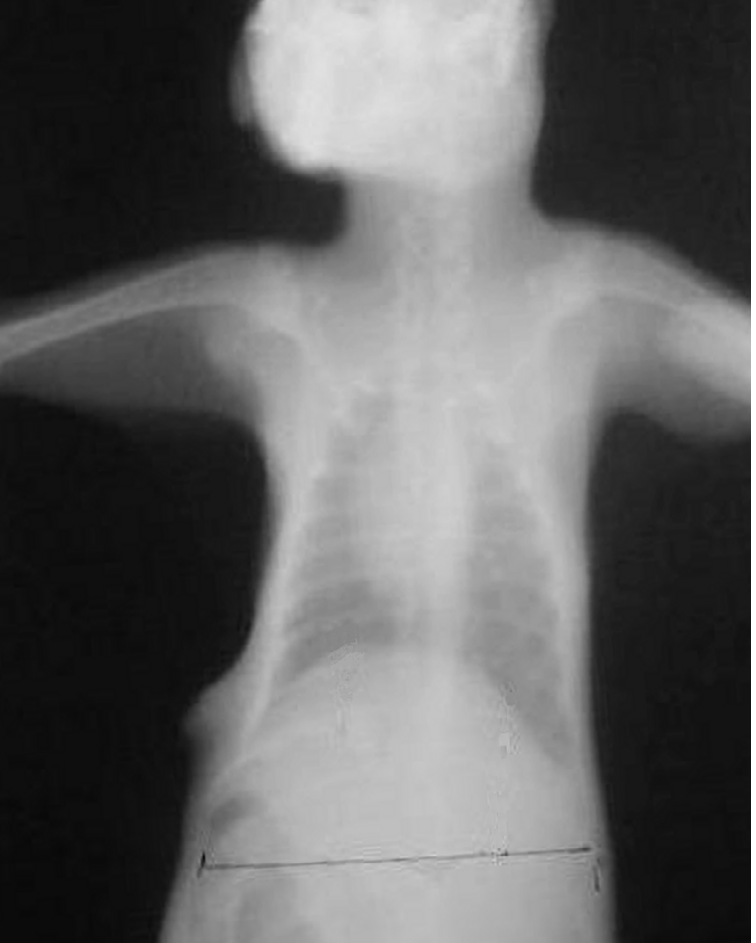
Postoperative 7^th ^day chest radiograph of a rabbit of BTX-A group.

**Figure 2:**
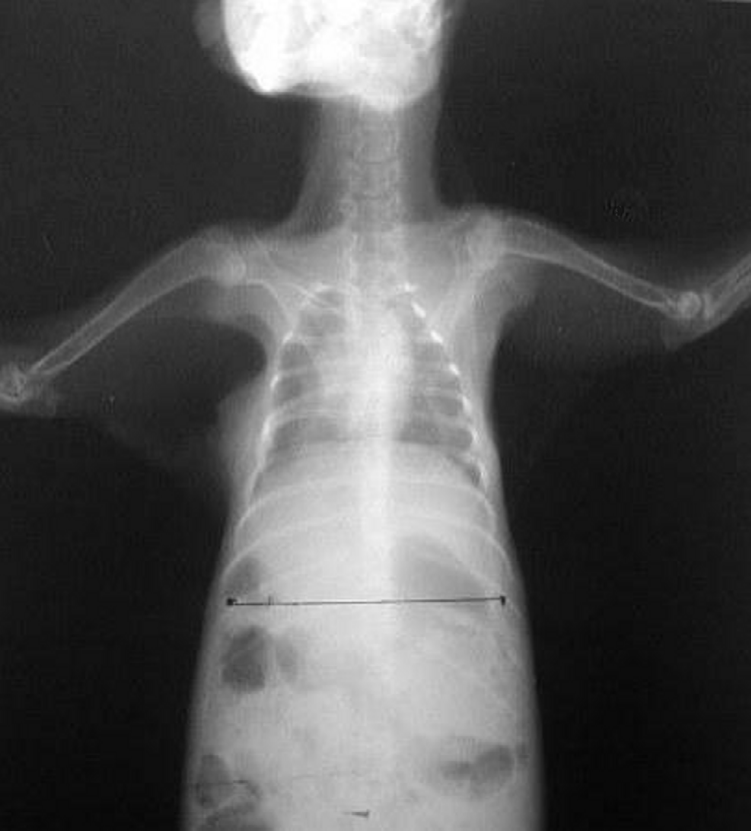
Postoperative 7^th ^day chest radiograph of a rabbit of control group.

The mean preoperative and postoperative 7^th ^day measurements of diaphragmatic heights are shown in Table 1 for both groups. The mean right hemidiaphragmatic elevations were 7.0 ± 2.5 in BTX-A (Figure 1) and 1.3 ± 1.2 in control (Figure 2) groups. Diaphragmatic elevations were significantly different (p = 0.0035) between groups. Fluoroscopic examination of diaphragmatic movements revealed that paralyses reversed in 8–12 weeks.

**Table 1:** Diaphragmatic heights, and diaphragmatic elevation differences in both groups (mean ± SD).

	Control group	BTX-A group	*P *value
Preop diaphragmatic heights (mm)	23.8 ± 5.4	24.8 ± 5.7	NS
Postop 7^th ^day diaphragmatic heights (mm)	25.2 ± 4.4	31.8 ± 4.3	0.045
Elevation difference (mm)	1.3 ± 1.2*	7.0 ± 2.4	0.0035

## Discussion

Some of the major factors delaying early hospital discharge following pulmonary lobectomy for lung cancer can be secondary to the adverse consequences of postlobectomy space. With increasing concern for quality of medical care, length of hospital stay, and cost of health care delivery, it has become the responsibility of the surgeon to control all factors that lead to a prolonged hospitalization.

Although several techniques have been used for prevention of postlobectomy space, optimal methods have not been described. Among these methods diaphragmatic elevation techniques have been used [1,2]. Phrenic nerve paralysis by crushing or local anaesthetic injection are some of the applied techniques for this purpose. Complication of permanent paralysis by crushing or ineffectiveness of short term paralysis by local anaesthetic injection reduces the usefulness of diaphragmatic elevation method. In general, diaphragmatic paralysis leads up to 20% loss of in pulmonary functions in adults with fully expanded normal lungs, in which elevated diaphragm will compress the expanded lung [6-8]. However when a patient had a lobectomy, diaphragmatic paralysis will not collapse an expanded lung, but instead obliterates a dead space.

In several rabbit studies, botulinum toxin A has been used as 5 to 10 units for relaxation of different muscles [9,10]. In the study of Aoki, the safety margin of BTX-A was determined as 13.9 ± 1.7 U/kg in mice [11]. As the duration of act of BTX is dose dependent, 10 U of BTX injection per animal was preferred into the diaphragm, which was safe and provided sufficient paralysis [12].

Botulinum toxin type A has an average clinical onset of action approximately 12 to 72 hours after injection, with a peak effect at one week. Then, plateau effect continues for 1 to 2 months [13]. For this reason, we took radiographs at the 3^rd ^and 7^th ^day for the evaluation of diaphragmatic elevation. We detected significant diaphragmatic elevation in the BTX-A group at day 7. Fluoroscopic examination of diaphragmatic movements revealed that paralysis reversed in 8–12 weeks. Our results are generally in concordance with these studies.

In this study, we showed that injection of BTX into the diaphragm can provide effective elevation for the prevention of postlobectomy space in an animal model.

Further studies are now required to evaluate its effectiveness in the clinical setting, particularly after pulmonary resection for lung cancer.

## References

[B1] Martini N, Ginsberg RJ, Pearson FG, Cooper JD, Deslauriers J, Ginsberg RJ, Hiebert CA, Patterson GA, Urschel HC (2002). Lobectomy. Thoracic Surgery.

[B2] Shields TW, Ponn RB, Shields TW, LoCicero III J, Ponn RB (2000). Complications of pulmonary resection. General Thoracic Surgery.

[B3] Okur E, Kir A, Halezeroglu S, Alpay AL, Atasalihi A (2001). Pleural tenting following upper lobectomies or bilobectomies of the lung to prevent residual air space and prolonged air leak. Eur J Cardiothorac Surg.

[B4] Cerfolio RJ, Holman WL, Katholi CR (2000). Pneumoperitoneum after concomitant resection of the right middle and lower lobes (bilobectomy). Ann Thorac Surg.

[B5] Graham HK, Aoki KR, Autti-Ramo I, Boyd RN, Delgado MR, Gaebler-Spira DJ, Gormley ME, Guyer BM, Heinen F, Holton AF, Matthews D, Molenaers G, Motta F, Garcia Ruiz PJ, Wissel J (2000). Recommendations for the use of botulinum toxin type A in the management of cerebral palsy. Gait Posture.

[B6] Shields TW, Shields TW, LoCicero III J, Ponn RB (2000). Diaphragmatic function, diaphragmatic paralysis and eventration of the Diaphragm. General Thoracic Surgery.

[B7] Urmey WF, McDonald M (1992). Hemidiaphragmatic paresis during interscalene brachial plexus block: effects on pulmonary function and chest wall mechanics. Anesth Analg.

[B8] Fell SC (1998). Surgical anatomy of the diaphragm and the phrenic nerve. Chest Surg Clin N Am.

[B9] Ohtsuki H, Hasebe S, Okano M, Furuse T (1998). Morphological changes in the orbital surface layer muscle of the rabbit eye produced by botulinum toxin. Ophthalmologica.

[B10] Kim HS, Hwang JH, Jeong ST, Lee YT, Lee PK, Suh YL, Shim JS (2003). Effect of muscle activity and botulinum toxin dilution volume on muscle paralysis. Dev Med Child Neurol.

[B11] Aoki KR (2002). Botulinum neurotoxin serotypes A and B preparations have different safety margins in preclinical models of muscle weakening efficacy and systemic safety. Toxicon.

[B12] Aoki KR, Guyer B (2001). Botulinum toxin type A and other botulinum toxin serotypes: a comparative review of biochemical and pharmacological actions. Eur J Neurol.

[B13] Tilton AH (2003). Injectable neuromuscular blockade in the treatment of spasticity and movement disorders. J Child Neurol.

